# Postprandial changes in gene expression of cholesterol influx and efflux mediators after intake of SFA compared with *n*-6 PUFA in subjects with and without familial hypercholesterolaemia: secondary outcomes of a randomised controlled trial

**DOI:** 10.1017/jns.2019.25

**Published:** 2019-08-13

**Authors:** Linn K. L. Øyri, Ingunn Narverud, Martin P. Bogsrud, Patrik Hansson, Lena Leder, Marte G. Byfuglien, Marit B. Veierød, Magne Thoresen, Stine M. Ulven, Kirsten B. Holven

**Affiliations:** 1 Department of Nutrition, Institute of Basic Medical Sciences, University of Oslo, Sognsvannsveien 9, 0372 Oslo, Norway; 2 Norwegian National Advisory Unit on Familial Hypercholesterolemia, Department of Endocrinology, Morbid Obesity and Preventive Medicine, Oslo University Hospital, Aker Hospital, Building 6, 6th floor, Trondheimsveien 232, 0586 Oslo, Norway; 3 Unit for Cardiac and Cardiovascular Genetics, Oslo University Hospital, Kirkeveien 166, 0450 Oslo, Norway; 4 Mills AS, Sofienberggt. 19, 0558 Oslo, Norway; 5 Oslo Centre for Biostatistics and Epidemiology, Department of Biostatistics, Institute of Basic Medical Sciences, University of Oslo, Sognsvannsveien 9, 0372 Oslo, Norway

**Keywords:** Familial hypercholesterolaemia, Fat quality, Gene expression, LDL receptor, Postprandial responses, *C*_*T*_, cycle threshold, FH, familial hypercholesterolaemia, LDL-C, LDL-cholesterol, LDLR, LDL receptor, PBMC, peripheral blood mononuclear cells, SREBP, sterol regulatory element binding protein

## Abstract

The long-term cholesterol-lowering effect of replacing intake of SFA with PUFA is well established, but has not been fully explained mechanistically. We examined the postprandial response of meals with different fat quality on expression of lipid genes in peripheral blood mononuclear cells (PBMC) in subjects with and without familial hypercholesterolaemia (FH). Thirteen subjects with FH (who had discontinued lipid-lowering treatment ≥4 weeks prior to both test days) and fourteen normolipidaemic controls were included in a randomised controlled double-blind crossover study with two meals, each with 60 g of fat either mainly SFA (about 40% energy) or *n*-6 PUFA (about 40% energy). PBMC were isolated in fasting, and 4 and 6 h postprandial blood samples. Expression of thirty-three lipid genes was analysed by reverse transcription quantitative PCR. A linear mixed model was used to assess postprandial effects between meals and groups. There was a significant interaction between meal and group for *MSR1* (*P* = 0·03), where intake of SFA compared with *n*-6 PUFA induced a larger reduction in gene expression in controls only (*P* = 0·01). Intake of SFA compared with *n*-6 PUFA induced larger reductions in gene expression levels of *LDLR* and *FADS1/2*, smaller increases of *INSIG1* and *FASN*, and larger increases of *ABCA1* and *ABCG1* (*P* = 0·01 for all, no group interaction). Intake of SFA compared with *n*-6 PUFA induced changes in gene expression of cholesterol influx and efflux mediators in PBMC including lower *LDLR* and higher *ABCA1/G1*, potentially explaining the long-term cholesterol-raising effect of a high SFA intake.

## Graphical abstract

**Figure:**
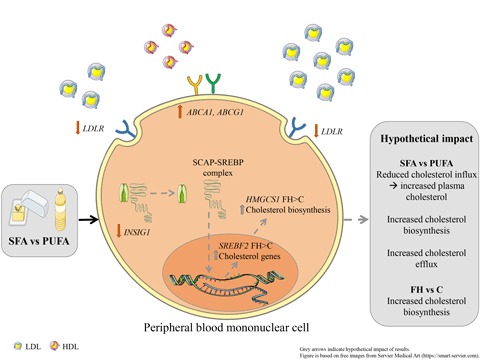

Elevated total cholesterol and LDL-cholesterol (LDL-C) concentrations are established risk factors for CVD^(1)^. In a meta-analysis of sixty controlled trials, Mensink *et al*.^(2)^ showed a significant decrease in serum LDL-C when SFA were replaced with unsaturated fatty acids. We previously showed that by exchanging only a few regularly consumed food items with less SFA and more PUFA for 8 weeks, serum total cholesterol and LDL-C significantly decreased in hypercholesterolaemic subjects^(3)^. For every energy percentage of SFA that is replaced with PUFA, a 2–3 % risk reduction is seen in CHD^(4)^, providing strong evidence for the role of fat quality in CVD development^(^^5^^,^^6^^)^.

Patients with familial hypercholesterolaemia (FH) are characterised by genetically elevated cholesterol levels, mainly due to a mutation in the gene coding for the LDL receptor (LDLR)^(7)^. Thus, these patients have increased CVD mortality^(8)^. It has also been suggested that subjects with FH may have an altered metabolism of TAG-rich lipoproteins^(^^9^^,^^10^^)^. We recently showed that the postprandial TAG response did not differ between young FH subjects and healthy controls after intake of high-fat meals rich in SFA or PUFA. However, the TAG peaked later after intake of SFA compared with PUFA^(11)^.

Peripheral blood mononuclear cells (PBMC) are circulating cells playing an important role in CVD development and are exposed to environmental factors such as dietary components^(12)^. Studies have shown that PBMC reflect hepatic regulation of cholesterol metabolism^(^^13^^–^^15^^)^. Thus, since tissue availability in human studies is very limited, PBMC may serve as a model system to investigate cholesterol metabolism.

The exact mechanisms behind the LDL-C-lowering effect of replacing SFA with PUFA are not fully explained, but may potentially be through regulation of the LDLR^(16)^. This should be further clarified in humans to strengthen the evidence for current dietary recommendations. If SFA induce a cholesterol-increasing effect through modulation of the LDLR, it may be hypothesised that intake of SFA may be particularly unfavourable for patients with FH and LDLR deficiency^(17)^. The aim of the present study was to explore the expression of lipid-related genes in PBMC after a single meal with high SFA *v.* high *n*-6 PUFA content in subjects with and without FH.

## Subjects and methods

### Subjects

The subjects and study design including inclusion and exclusion criteria have been described in detail previously^(11)^. Briefly, in this randomised controlled double-blind crossover study we included two groups, one with genetically verified heterozygous FH subjects and one with normolipidaemic controls, both aged 18–30 years. The subjects were included if they had BMI 18·5–30·0 kg/m^2^, C-reactive protein levels ≤10 mg/l, TAG ≤4 mmol/l and no metabolic co-morbidities. An additional inclusion criterion for the FH subjects was the presence of a FH mutation in the gene encoding the LDLR. All FH subjects were treated with lipid-lowering medications, but discontinued the treatment during the last 4 weeks prior to the first test day and during the whole period between the first and second test day.

### Study design

**Fig. 1.**
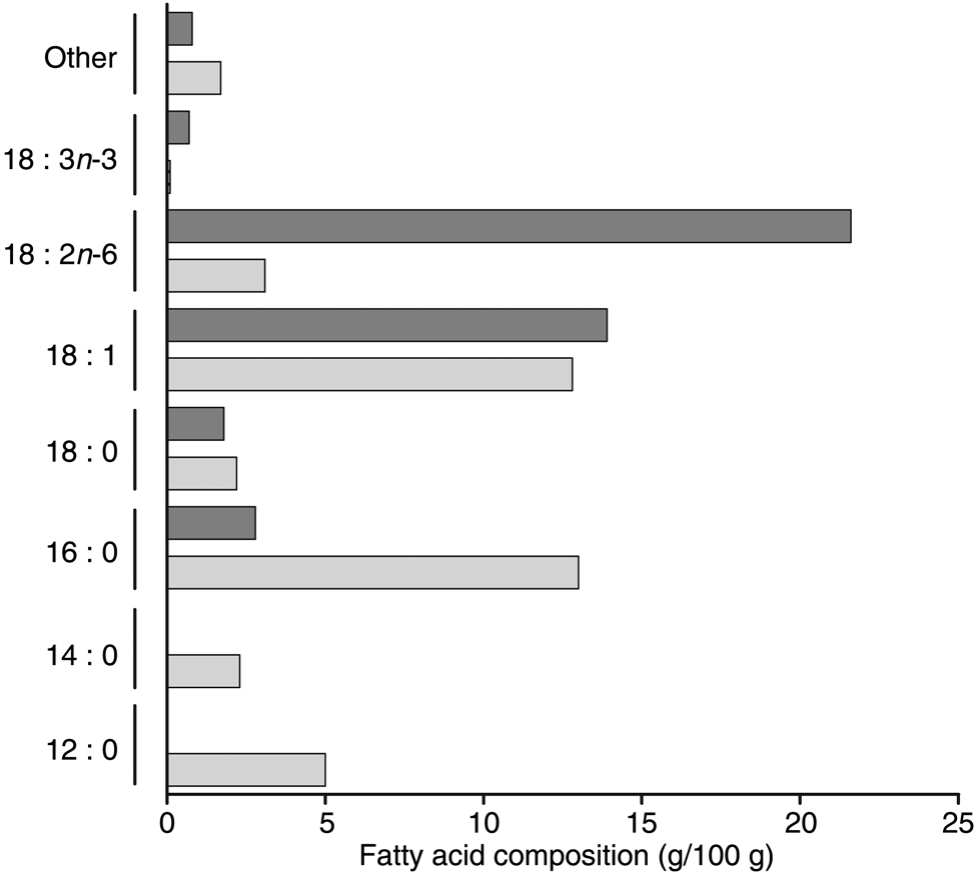
Fatty acid composition of the test meals. 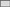, SFA meal; 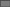, PUFA meal.

The FH subjects and normolipidaemic controls ingested two meals with different fat quality in a randomised order with a wash-out period of 3–5 weeks between the meals. The two meals (150 g) were high in fat (60 g; 70 % energy) and with either mainly SFA (about 40 % energy) or *n*-6 PUFA (about 40 % energy), and were blinded to the participants and care providers. The two meals contained the same amount of energy, MUFA, carbohydrates and proteins. Fat originated from palm oil and coconut oil in the SFA meal, and from sunflower-seed oil and rapeseed oil in the *n*-6 PUFA meal. The fatty acid composition of the meals is illustrated in Fig. 1. Venous blood samples were taken after 12 h of fasting (baseline, 0 h) and 4 and 6 h after meal consumption. The study visits were performed at the University of Oslo, Norway between March and May 2016. This study was conducted according to the guidelines laid down in the Declaration of Helsinki and all procedures involving human subjects were approved by the Regional Committees for Medical and Health Research Ethics (REK 2015/2392/REK sør-øst B). Written informed consent was obtained from all subjects. The study was registered at http://www.ClinicalTrials.gov (registration no. NCT02729857). The main results from the study have been published previously^(11)^. This paper presents pre-specified secondary outcomes from the study.

### Routine measures

Serum was collected from silica gel tubes (Becton Dickenson Vacutainer Systems) and stored at room temperature for 30–60 min until centrifugation (1500 ***g***; 15 min). Whole blood samples in EDTA tubes (Becton Dickenson Vacutainer Systems) were kept at room temperature until analysed. Standard blood chemistry was measured in serum and whole blood using routine laboratory methods at an accredited medical laboratory (Fürst Medical Laboratory).

### Gene expression analysis in peripheral blood mononuclear cells

PBMC were isolated from blood using BD Vacutainer Cell Preparation tubes with sodium heparin according to the manufacturer (Becton Dickinson) and stored as pellets at −80°C until further RNA isolation. Total RNA was isolated using an RNeasy mini kit (Qiagen), and treated with DNase I (Qiagen) according to the manufacturer's instructions and stored at −80°C. The quantity and quality of RNA were measured using an ND 1000 Spectrophotometer (Saveen Werner Carlson Circle) and an Agilent Bioanalyser (Agilent Technology), respectively. A high-capacity RNA-to-cDNA kit (catalogue no. 4387406; Applied Biosystems) was used to reverse transcribe 500 ng of RNA from all samples. Quantitative real-time PCR was performed on an ABI PRISM 7900HT Sequence Detector System (Applied Biosystems) using custom TaqMan array cards (Life Technologies). Acyl-CoA synthetase long-chain family member (ACSL) 3 and ACSL4 were chosen as reference genes based on the lowest between-sample variation as recommended by the manufacturer's protocol. For this purpose and to calculate the relative mRNA expression level for each transcript, the software ExpressionSuite v1.1 (ThermoFisher Scientific) was used. Further, the ΔΔ cycle threshold (*C*_*T*_) method^(18)^ was used. Briefly, the *C*_*T*_ values of each target gene were normalised to the C_*T*_ values of the two reference genes (=Δ*C*_*T*_). ΔΔ*C*_*T*_ was then calculated as Δ*C*_*T, 4 or 6 h*_ minus Δ*C*_*T, baseline (0 h)*_. The fold change in mRNA expression was calculated as 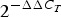.

An overview of the lipid-related genes, respective metabolic pathway and assay identification number of the inventoried TaqMan probe and primer sets used is provided in Table 1.

**Table 1.** Overview of the lipid-related genes examined by quantitative real-time PCR

Gene symbol	Full name	Function	ThermoFisher Scientific's assay no.
*ACAT1*	Acetyl-CoA acetyltransferase 1	Cholesterol biosynthesis	Hs01011096_m1
*DHCR24*	24-Dehydrocholesterol reductase	Cholesterol biosynthesis	Hs00207388_m1
*DHCR7*	7-Dehydrocholesterol reductase	Cholesterol biosynthesis	Hs01023087_m1
*FDFT1*	Squalene synthase	Cholesterol biosynthesis	Hs00926054_m1
*FDPS*	Farnesyl diphosphate synthase	Cholesterol biosynthesis	Hs01578769_g1
*HMGCR*	3-Hydroxy-3methylglutaryl-CoA reductase	Cholesterol biosynthesis	Hs00168352_m1
*HMGCS1*	3-Hydroxy-3-methylglutaryl-CoA synthase 1	Cholesterol biosynthesis	Hs00940429_m1
*INSIG1*	Insulin-induced gene 1	Cholesterol biosynthesis	Hs01650979_m1
*LSS*	Lanosterol synthase	Cholesterol biosynthesis	Hs01552331_m1
*SCAP*	Sterol regulatory element binding cleavage activating protein	Cholesterol biosynthesis	Hs00378725_m1
*CPT1A*	Carnitine palmitoyltransferase 1A	Fatty acid metabolism: β-oxidation	Hs00912671_m1
*CPT2*	Carnitine palmitoyltransferase 2	Fatty acid metabolism: β-oxidation	Hs04188816_m1
*ECI1*	Enoyl-CoA delta isomerase 1	Fatty acid metabolism: β-oxidation	Hs00157239_m1
*SLC25A20*	Solute carrier family 25 member 20	Fatty acid metabolism: β-oxidation	Hs00386383_m1
*ACSL1*	Acyl-CoA synthetase long-chain family member 1	Fatty acid metabolism: intracellular transport	Hs00960561_m1
*FADS1*	Fatty acid desaturase 1	Fatty acid metabolism: long-chain PUFA pathway	Hs00203685_m1
*FADS2*	Fatty acid desaturase 2	Fatty acid metabolism: long-chain PUFA pathway	Hs00927433_m1
*ACACA*	Acetyl-CoA carboxylase α	Fatty acid metabolism: synthesis	Hs01046047_m1
*FASN*	Fatty acid synthase	Fatty acid metabolism: synthesis	Hs01005622_m1
*SCD*	Stearoyl-CoA desaturase	Fatty acid metabolism: synthesis	Hs01682761_m1
*SORL1*	Sortilin related receptor 1	Lipoprotein metabolism	Hs00268342_m1
*LDLR*	LDL receptor	Lipoprotein metabolism	Hs01092524_m1
*MYLIP*	Myosin regulatory light chain interacting protein	Lipoprotein metabolism	Hs00203131_m1
*SORT1*	Sortilin-1	Lipoprotein metabolism	Hs00361760_m1
*VLDLR*	Very low-density lipoprotein receptor	Lipoprotein metabolism	Hs01045922_m1
*ABCA1*	ATP-binding cassette, sub family A, member 1	Reverse cholesterol transport	Hs01059118_m1
*ABCG1*	ATP-binding cassette, sub family G, member 1	Reverse cholesterol transport	Hs00245154_m1
*SCARB1*	Scavenger receptor class B member 1	Reverse cholesterol transport	Hs00969821_m1
*CD36*	CD 36 molecule	Scavenger receptor	Hs00169627_m1
*MSR1*	Macrophage scavenger receptor 1	Scavenger receptor	Hs00234007_m1
*NR1H3*	Nuclear receptor subfamily 1 group H member 3	Transcription factor targeting lipid genes	Hs00172885_m1
*SREBF1*	Sterol regulatory element binding transcription factor 1	Transcription factor targeting lipid genes	Hs01088691_m1
*SREBF2*	Sterol regulatory element binding transcription factor 2	Transcription factor targeting lipid genes	Hs01081778_m1

### Statistics

Subject characteristics are presented as medians and 25th–75th percentiles, or as frequencies and percentages. The Mann−Whitney test and χ^2^ test were used to compare subject characteristics and baseline (0 h) gene expression levels (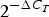) between FH and control subjects. We performed a linear mixed-model analysis on the changes from baseline (0 h) to 4 and 6 h (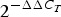 for time 4 and 6 h, respectively). Meal (i.e. change after the SFA *v.* PUFA meal), group (i.e. change in FH *v.* control subjects), time (i.e. change at 4 h *v.* 6 h from 0 h) and period (order of the meals) were included in the model. We tested for the following two-way interactions between the variables, one at a time: time–meal (i.e. difference in change from 4 to 6 h between the SFA and the PUFA meal), time–group (i.e. difference in change from 4 to 6 h in FH *v.* control subjects) and meal–group (i.e. difference in change from 0 h after the SFA and PUFA meal between FH and control subjects). Non-significant period or interaction effects were not included in the final models. Data were stratified by group and meal when there was a significant interaction effect. Normality, outliers and systematic trends of the residuals were examined in histograms and Q−Q plots to assess the adequacy of the fitted models. The maximum number of observations (indicated by *n* in the tables) included in the analysis was 108 (27 subjects × 2 meals × 2 times (4 and 6 h)). The postprandial effects are presented as means and standard errors of 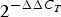 values. The Benjamini−Hochberg procedure (false discovery rate correction) was used to correct for the number of genes tested (*n* 33) for all variables in the linear mixed-model analysis, and Benjamini−Hochberg adjusted *P* values are presented. For genes that were differentially expressed between the meals, Spearman's rank-correlation coefficient (*r*) was estimated for change in gene expression and circulating lipids from 0 to 4 h with both groups combined. *P* values <0·05 were considered significant. Statistical analyses were conducted with SPSS version 24.0 and Benjamini−Hochberg adjustment was performed in Excel.

## Results

### Subject characteristics

Characteristics of the thirteen subjects with FH and the fourteen control subjects are presented in Table 2. There was no significant difference in age, sex and BMI between the groups^(11)^. Subjects with FH had significantly higher total cholesterol and LDL-C levels compared with controls^(11)^. There was no significant difference in the postprandial response (incremental AUC) of TAG, total cholesterol or LDL-C between meals or groups as previously shown^(11)^. At baseline (0 h), FH compared with control subjects had significantly lower expression of genes involved in fatty acid metabolism (*ACACA*, *CPT1A* and *FADS1*), cholesterol biosynthesis (*FDPS*), the gene coding for the scavenger receptor *MSR1* and genes involved in the transcription of lipid genes (*NR1H3* and *SREBF2*) (0·001 ≤ *P* ≤ 0·02) (Supplementary Table S1). There was no significant difference in the postprandial response of percentage distribution of plasma total SFA, MUFA, *n*-6 PUFA and *n*-3 PUFA between meals or groups (0·10 ≤ *P* ≤ 0·76; data not shown).

**Table 2.** Subject characteristics (Medians and 25th–75th percentiles; percentages)

	FH (*n* 13)				Controls (*n* 14)				
	SFA meal		PUFA meal		SFA meal		PUFA meal		
	Median	25th–75th percentiles	Median	25th–75th percentiles	Median	25th–75th percentiles	Median	25th–75th percentiles	P*
Age (years)									0·76
Median	25·0				24·5				
25th–75th percentiles	21·0–28·5				23·0–28·0				
Female (%)	61·5				64·3				1·0
BMI (kg/m^2^)									0·24
Median	22·9				22·1				
25th–75th percentiles	21·5–25·3				20·5–23·8				
TAG (mmol/l)	1·3	0·8–1·7	0·9	0·7–1·3	0·8	0·7–1·0	0·8	0·6–1·2	0·08
Total cholesterol (mmol/l)	7·2	6·5–8·7	7·9	6·5–9·0	4·1	3·9–4·4	4·1	3·6–4·5	<0·001
LDL-cholesterol (mmol/l)	5·7	5·3–7·4	6·3	5·4–7·5	2·4	1·8–2·9	2·3	1·8–2·8	<0·001
Neutrophils (%)	44	41–55	49	41–56	47	43–52	47	43–58	0·98
Lymphocytes (%)	40	34–46	37	33–43	40	35–43	38	27–44	0·87
Monocytes (%)	9	7–11	10	8–12	10	8–12	9	7–11	0·46
Eosinophils (%)	2	2–3	3	2–5	3	2–6	2	2–5	0·62
Basophils (%)	0	0–1	2	0–2	0	0–2	0	0–2	0·26

FH, familial hypercholesterolaemia.

* P values for group differences (mean of the two visits) from the Mann−Whitney test or χ^2^ test.

### Postprandial responses in peripheral blood mononuclear cell gene expression levels

We found no significant effect of period (order of the meals; 0·10 ≤ *P* ≤ 0·95) and no significant interaction between time and meal (0·73 ≤ *P* ≤ 0·92) and between time and group (0·98 ≤ *P* ≤ 0·99), thus these are not included in the final model. There were no significant interactions between meal and group (0·19 ≤ *P* ≤ 0·95), except for the scavenger receptor *MSR1* (*P* = 0·03), where intake of SFA compared with *n*-6 PUFA induced a larger reduction in gene expression in controls only (*P* = 0·01, Table 3). Significant differences between meals were found for seven out of thirty-three genes, independent of group. Intake of SFA compared with *n*-6 PUFA induced larger reductions in expression levels of *LDLR* (Table 3) and genes involved in fatty acid desaturation (*FADS1*, *FADS2*; Table 3), and smaller increases in expression levels of genes involved in cholesterol biosynthesis (*INSIG1*; Table 3) and fatty acid synthesis (*FASN*; Table 3) (*P* = 0·01 for all). Moreover, intake of SFA compared with *n*-6 PUFA induced larger increases in expression levels of genes involved in reverse cholesterol transport (*ABCA1*, *ABCG1*; *P* = 0·01 for both; Table 3).

**Table 3.** Postprandial changes in gene expression in peripheral blood mononuclear cells that were significantly different between meals or groups* (Mean values with their standard errors)

		SFA				PUFA					
		4 h (2^−ΔΔ*CT*^)		6 h (2^−ΔΔ*CT*^)		4 h (2^−ΔΔ*CT*^)		6 h (2^−ΔΔ*CT*^)			
Target gene	*n*	Mean	se	Mean	se	Mean	se	Mean	se	*P*_meal_	*P*_group_
*ABCA1*	98									0·01†	0·44
FH		1·75	0·18	1·62	0·19	1·45	0·16	1·29	0·13		
C		1·57	0·13	1·29	0·08	1·20	0·09	1·25	0·17		
*ABCG1*	98									0·01†	0·73
FH		1·47	0·13	1·42	0·14	1·27	0·04	1·22	0·11		
C		1·57	0·14	1·37	0·11	1·30	0·13	0·92	0·10		
*CPT1A*	95									0·42	0·01§
FH		1·32	0·15	2·11	0·25	1·24	0·09	1·67	0·11		
C		0·89	0·12	1·32	0·10	1·03	0·08	1·30	0·07		
*FADS1*	104									0·01‡	0·12
FH		0·93	0·05	0·90	0·06	1·01	0·10	1·02	0·09		
C		0·71	0·04	0·70	0·04	1·02	0·10	0·89	0·07		
*FADS2*	104									0·01‡	0·01§
FH		1·06	0·08	1·13	0·11	1·30	0·13	1·13	0·08		
C		0·72	0·08	0·80	0·07	1·03	0·10	1·00	0·07		
*FASN*	96									0·01‡	0·15
FH		1·13	0·10	1·26	0·14	1·34	0·11	1·30	0·09		
C		0·95	0·06	1·07	0·06	1·16	0·09	1·17	0·06		
*HMGCS1*	100									0·23	0·01§
FH		1·30	0·10	1·14	0·10	1·14	0·08	1·20	0·12		
C		0·81	0·03	0·81	0·07	1·09	0·09	0·97	0·05		
*INSIG1*	94									0·01‡	0·55
FH		1·19	0·06	1·01	0·07	1·26	0·11	1·26	0·11		
C		1·11	0·08	0·96	0·07	1·15	0·07	1·13	0·07		
*LDLR*	95									0·01‡	0·44
FH		0·78	0·05	0·84	0·11	0·99	0·08	0·89	0·06		
C		0·76	0·07	0·74	0·08	1·21	0·09	1·05	0·08		
*MSR1*	104									C 0·01‡, FH 0·26	SFA 0·15, PUFA 0·37
FH		1·06	0·11	1·03	0·10	0·90	0·07	0·85	0·06		
C		0·81	0·04	0·87	0·06	1·09	0·10	1·07	0·10		
*SREBF2*	98									0·22	0·02§
FH		1·10	0·11	1·03	0·11	1·06	0·08	1·10	0·09		
C		0·80	0·07	0·67	0·04	0·89	0·04	0·80	0·04		

*C*_*T*_, cycle threshold; *n*, number of subjects × 2 meals × 2 times (4 and 6 h); *P*_meal_, *P* value for change after the SFA *v*. PUFA meal; *P*_group_, *P* value for change in FH *v*. control subjects; *ABCA1*, ATP-binding cassette, subfamily A, member 1; FH, familial hypercholesterolaemia; C, control; *ABCG1*, ATP-binding cassette, subfamily G, member 1; *CPT1A*, carnitine palmitoyltransferase 1A; *FADS1*, fatty acid desaturase 1; *FADS2*, fatty acid desaturase 2; *FASN*, fatty acid synthase; *HMGCS1*, 3-hydroxy-3-methylglutaryl-CoA synthase 1; *INSIG1*, insulin-induced gene 1; *LDLR*, LDL receptor; *MSR1*, macrophage scavenger receptor 1; *SREBF2*, sterol regulatory element binding transcription factor 2.

* Data are presented as fold change from baseline (0 h) and reference genes (*ACSL3* and *ACSL4*) (2^−ΔΔCT^). *P* values are Benjamini−Hochberg adjusted *P* values from a linear mixed model. There was a significant interaction between meal and group for *MSR1* (*P* = 0·03); thus, *P* values are presented stratified by group and meal.

† SFA > PUFA.

‡ SFA < PUFA.

§ FH > control.

FH compared with control subjects had significantly larger postprandial increases in expression levels of genes involved in β-oxidation (*CPT1A*), fatty acid desaturation (*FADS2*), cholesterol biosynthesis (*HMGCS1*) and transcription of lipid genes (*SREBF2*), independent of meal (0·01 ≤ *P* ≤ 0·02; Table 3). Postprandial expression of genes that did not change significantly different between meals or groups is presented in Supplementary Table S2.

Furthermore, we correlated the change from 0 to 4 h (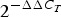) in expression of the genes that were significantly different between meals (*ABCA1*, *ABCG1*, *FADS1*, *FADS2*, *FASN*, *INSIG1*, *LDLR* and *MSR1*) with the change from 0 to 4 h in circulating LDL-C and TAG levels. However, there were no significant correlations between the changes in gene expression of the selected genes and circulating LDL-C and TAG levels after any of the two meals (−0·26 ≤ *r* ≤ 0·39; 0·05 ≤ *P* ≤ 0·96; data not shown).

## Discussion

In the present study, we found that intake of SFA compared with *n*-6 PUFA modulated the expression of several key genes in lipid metabolism including a larger reduction of *LDLR*, smaller increase of *INSIG1* and larger increases of *ABCA1* and *ABCG1* in PBMC. These effects may contribute to the explanation of some of the unfavourable effects induced by SFA compared with *n*-6 PUFA intake on circulating cholesterol levels.

Intake of SFA has consistently been shown to increase circulating cholesterol levels in dietary intervention studies^(2,3,6)^. However, the molecular mechanisms remain to be completely understood. Few studies have investigated the impact of fat quality on *LDLR* gene expression in humans. In the present study, a larger reduction in postprandial gene expression level of *LDLR* in PBMC was found after intake of SFA compared with *n*-6 PUFA. Decreased *LDLR* gene expression has also been shown in a previous postprandial study after SFA *v.* MUFA intake^(19)^. Long-term human studies exploring gene expression in PBMC have shown increased *LDLR* expression after replacing intake of SFA with PUFA^(20)^, increased *LDLR* expression after decreasing intake of SFA^(16)^ and decreased *LDLR* expression after increasing intake of SFA^(21)^. Recently, also a tendency towards lower PBMC gene expression of *LDLR* was observed after 3 weeks of a low-carbohydrate/high-fat diet (*P* = 0·06)^(22)^. Moreover, previous results in animals show that PUFA up-regulate LDLR protein and mRNA levels, and that SFA decrease LDLR activity, protein and mRNA abundance and alter LDL composition and size^(^^17^^,^^23^^–^^25^^)^. Collectively, these studies suggest that high intake of SFA affect the gene expression of *LDLR*, which may, at least partly, explain the established long-term cholesterol-raising effect of an SFA-rich diet.

Since a significant down-regulation of *LDLR* was seen already 4–6 h after a single meal rich in SFA compared with *n*-6 PUFA, this unfavourable effect may potentially be even larger if meals rich in SFA are ingested several times daily for a longer period of time. The short duration of the study may explain why we did not observe any postprandial change in LDL-C levels after any of the meals in either the FH or control group^(11)^. Thus, the impact on *LDLR* gene expression should be investigated in larger long-term studies where SFA are replaced with *n*-6 PUFA in order to elucidate mechanisms supporting the current nutritional recommendations of replacing intake of SFA with PUFA^(26)^. Furthermore, many FH subjects are characterised by a reduced number of functional LDLR. Thus, if intake of meals rich in SFA further reduces LDLR expression, dietary fat quality could in the long run have an even greater impact on circulating LDL-C levels in persons with FH than in normolipidaemic individuals.

The effect of SFA compared with PUFA on *LDLR* was accompanied by a smaller increase in gene expression of *INSIG1*, an important factor in the sterol regulatory element binding cleavage activating protein–sterol regulatory element binding protein (SCAP–SREBP) regulation of cholesterol homeostasis. The INSIG–SCAP–SREBP complex serves as an intracellular sterol sensor, where SREBP is a transcription factor targeting genes involved in intracellular cholesterol homeostasis^(^^27^^,^^28^^)^. In an intracellular cholesterol-deprived state, e.g. when LDLR is reduced, the SCAP–SREBP complex dissociates from INSIG1, and SREBP is transferred to the nucleus for transcription of its target genes. Thus, the combination of low gene expression of *LDLR* and *INSIG1* may lead to increased intracellular cholesterol production.

In line with others, we found a significantly larger increase in the gene expression of *ABCA1* and *ABCG1* after intake of SFA compared with PUFA^(29)^. This has been found by others after intake of SFA compared with MUFA^(19)^. ABCA1 and ABCG1 are known to play an important role in the cholesterol efflux from macrophages to HDL^(30,31)^. Thus, our results may indicate an increased cholesterol efflux possibly due to INSIG-induced intracellular cholesterol production, which may lead to increased HDL levels. The physiological explanations behind these changes in gene expressions are not understood. However, since cholesterol is an important membrane component^(32)^ and the fatty acid content of phospholipids is prone to dietary changes^(33)^, the present changes in gene expression may play a role in membrane stability.

As expected, we found larger reduction in expression levels of genes involved in fatty acid desaturation (*FADS1*, *FADS2*) and smaller increase in expression level of a gene involved in fatty acid synthesis (*FASN*) after the SFA-rich compared with the *n*-6 PUFA-rich meal. This finding is in line with previous studies^(^^34^^–^^36^^)^. Furthermore, intake of SFA compared with *n*-6 PUFA induced a larger reduction in the gene expression of *MSR1* in controls only. *MSR1* encodes the scavenger receptor protein SR-A1 which has been shown to have a role in atherosclerosis by mediating uptake of modified LDL (primarily acetylated LDL); however, the underlying mechanisms are not yet fully elucidated. Recent evidence also points to important roles for SR-A1 in inflammation and innate immunity^(37)^. Thus SR-A1 has been suggested to have either anti-atherogenic or pro-atherogenic effects^(37)^. The expression of MSR1 has been suggested to be differently modulated under different genetic backgrounds and during macrophage differentiation^(37)^. The expression of the scavenger receptors CD36 and SR-A1 are regulated by the nuclear receptor PPARγ. PUFA are known ligands of PPARγ; thus, one may speculate that PUFA might regulate SR-A1 through a PPAR mechanism, potentially explaining the larger reduction in expression after intake of SFA compared with *n*-6 PUFA in healthy controls^(38)^.

Post-transcriptional alterations in LDLR may potentially explain the lower number of functional LDLR and hence the higher circulating level of LDL-C in FH patients^(7)^, and may also explain the similar baseline gene expression level of *LDLR* observed among subjects with and without FH in this study. Furthermore, we found lower baseline expression levels of genes involved in cholesterol biosynthesis (*FDPS*) and transcription of lipid genes (*NR1H3* and *SREBF2*) in FH subjects compared with controls. However, there was a larger postprandial increase in the expression of genes involved in cholesterol biosynthesis (*HMGCS1*) and transcription of lipid genes (*SREBF2*) in FH subjects compared with controls. Thus, the postprandial changes in *HMGCS1* and *SREBF2* may result in increased cholesterol biosynthesis in FH subjects compared with controls.

This is a double-blind, randomised and controlled crossover study, presenting secondary outcomes defined prior to study initiation. A major limitation of the study is the relatively low number of subjects, albeit in line with previous similar studies^(^^19^^,^^29^^,^^39^^)^. The clinical relevance of the findings is at present not clear due to the explorative nature of the study, thus larger studies are needed. Another limitation of this study, and most human studies, is that liver biopsies are not readily available and we therefore have to extrapolate results obtained from PBMC. However, PBMC have been shown to be a good model system reflecting hepatic regulation of cholesterol metabolism^(^^13^^–^^15^^)^.

**Fig. 2.**
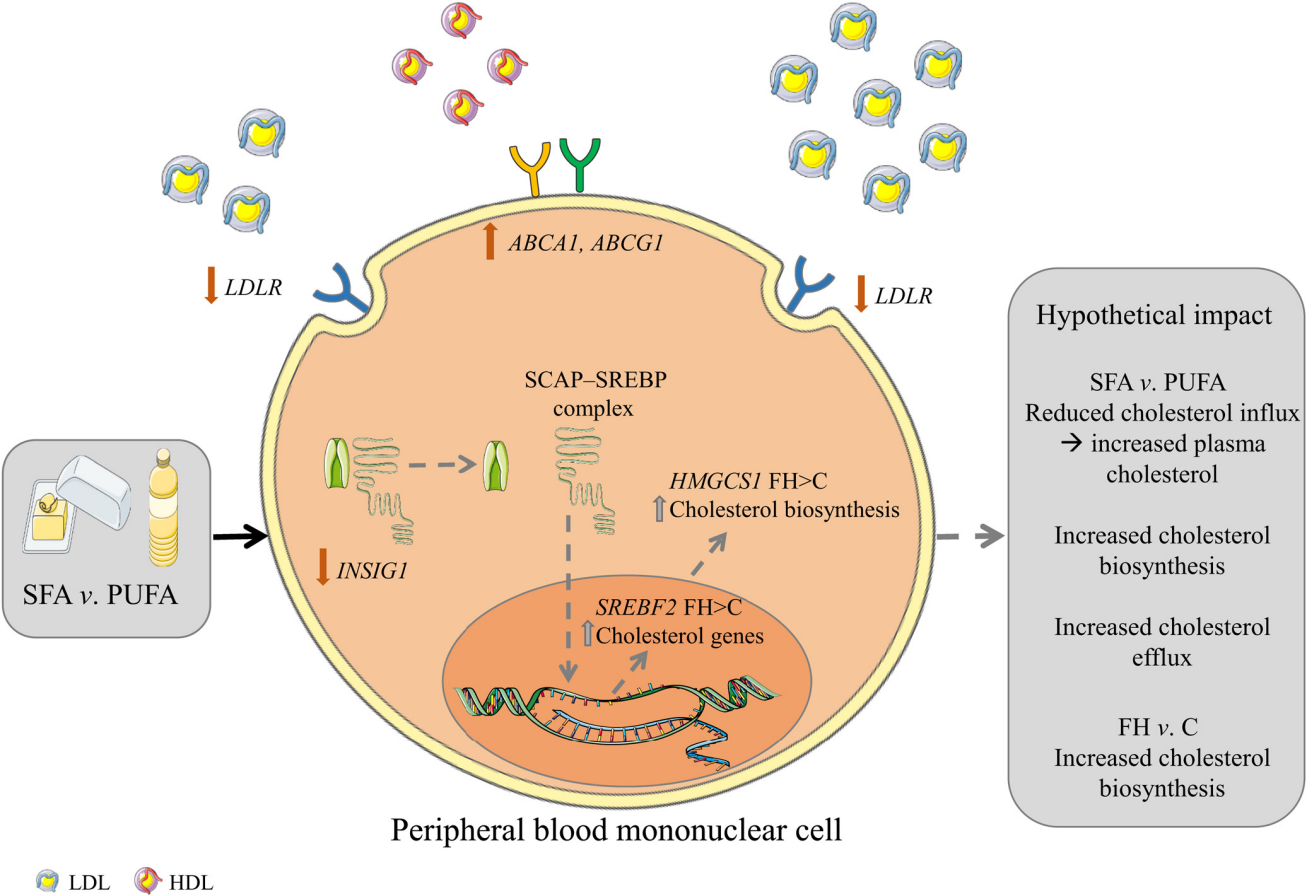
Graphical summary. Hypothetical impact of postprandial gene expression in cholesterol homeostasis after SFA *v. n*-6 PUFA intake. Intake of SFA *v. n*-6 PUFA induces a larger reduction in the gene expression of LDL receptor (*LDLR*) and a lower increase of insulin-induced gene 1 (*INSIG1*) which in combination may potentially result in decreased cholesterol influx, increased circulating cholesterol and increased cholesterol biosynthesis. Furthermore, intake of SFA *v. n*-6 PUFA induces larger increases in the gene expression of ATP-binding cassette, subfamily A, member 1 (*ABCA1*) and ATP-binding cassette, subfamily G, member 1 (*ABCG1*) which may potentially result in increased cholesterol efflux. Grey arrows indicate hypothetical impact of results. SCAP, sterol regulatory element binding cleavage activating protein; SREBP, sterol regulatory element binding protein; *HMGCS1*, 3-hydroxy-3-methylglutaryl-CoA synthase 1; FH, familial hypercholesterolaemia; C, control; *SREBF2*, sterol regulatory element binding transcription factor 2. The figure is based on free images from ServierMedical Art (https://smart.servier.com).

Intake of SFA compared with *n*-6 PUFA induced changes in gene expression of cholesterol influx and efflux mediators in PBMC including a larger reduction of *LDLR* and larger increases of *ABCA1/G1*, possibly, at least partly, explaining some of the cholesterol-raising effects of a high SFA intake (for a graphical summary, see Fig. 2). These data support the current dietary guidelines of replacing SFA with *n*-6 PUFA and underscore the importance of recommending reduced SFA intake in FH patients with less functional LDLR.

## Supplementary material

For supplementary material accompanying this paper visit https://doi.org/10.1017/jns.2019.25. click here to view supplementary material
