# Supplementary material for: Postprandial changes in gene expression of cholesterol influx and efflux mediators after intake of SFA compared with n-6 PUFA in subjects with and without familial hypercholesterolaemia: secondary outcomes of a randomised controlled trial
Source: J Nutr Sci. 2019 Aug 13;8:e27. doi: 10.1017/jns.2019.25 (PMC6692810; doi:10.1017/jns.2019.25)
Supplement: Supplementary file 1 [file S2048679019000259sup001.docx]

**Supplementary Table 1.** Gene expression in peripheral blood mononuclear cells at baseline (0 h) in the two groups.

|  | **FH*, n* = 13**  **(**2^-∆^*^CT^***)** |  | **Controls, *n* = 14**  **(**2^-∆^*^CT^***)** | ***p*** |
| --- | --- | --- | --- | --- |
| *ABCA1* | 0.19 (0.16-0.26) |  | 0.23 (0.19-0.32) | 0.24 |
| *ABCG1* | 0.38 (0.33-0.41) |  | 0.39 (0.30-0.42) | 0.98 |
| *ACACA* | 0.21 (0.18-0.27) |  | 0.27 (0.24-0.32) | **0.01** |
| *ACAT1* | 1.10 (0.96-1.20) |  | 1.13 (1.03-1.34) | 0.49 |
| *ACSL1* | 1.09 (0.97-1.23) |  | 1.23 (0.98-1.47) | 0.12 |
| *CD36* | 13.2 (11.8-15.1) |  | 13.8 (11.2-14.7) | 0.94 |
| *CPT1A* | 1.96 (1.57-2.41) |  | 2.68 (2.39-3.13) | **0.002** |
| *CPT2* | 1.10 (1.01-1.18) |  | 1.14 (1.07-1.21) | 0.40 |
| *DHCR24* | 0.45 (0.37-0.57) |  | 0.49 (0.46-0.53) | 0.26 |
| *DHCR7* | 1.10 (0.88-1.16) |  | 1.05 (0.92-1.12) | 0.76 |
| *ECI1* | 1.42 (1.24-1.52) |  | 1.46 (1.27-1.59) | 0.28 |
| *FADS1* | 0.54 (0.48-0.60) |  | 0.68 (0.58-0.78) | **0.01** |
| *FADS2* | 0.47 (0.40-1.53) |  | 0.58 (0.47-1.19) | 0.28 |
| *FASN* | 2.13 (2.04-2.27) |  | 2.24 (1.94-2.45) | 0.53 |
| *FDFT1* | 0.55 (0.44-0.57) |  | 0.59 (0.48-0.90) | 0.16 |
| *FDPS* | 0.83 (0.79-1.09) |  | 1.14 (0.95-1.44) | **0.01** |
| *HMGCR* | 1.38 (1.32-1.55) |  | 1.41 (1.36-1.54) | 0.62 |
| *HMGCS1* | 0.35 (0.32-0.41) |  | 0.37 (0.33-0.42) | 0.33 |
| *INSIG1* | 1.62 (1.44-1.77) |  | 1.73 (1.34-1.82) | 0.65 |
| *LDLR* | 0.74 (0.52-0.86) |  | 0.77 (0.61-0.87) | 0.33 |
| *LSS* | 2.52 (2.22-2.99) |  | 2.82 (2.52-3.13) | 0.13 |
| *MSR1* | 0.42 (0.23-0.58) |  | 0.71 (0.37-0.90) | **0.02** |
| *MYLIP* | 0.55 (0.49-0.68) |  | 0.49 (0.44-0.58) | 0.09 |
| *NR1H3* | 0.22 (0.20-0.27) |  | 0.30 (0.26-0.32) | **0.01** |
| *SCAP* | 2.41 (2.22-2.72) |  | 2.52 (2.32-2.77) | 0.58 |
| *SCARB1* | 0.28 (0.25-0.33) |  | 0.28 (0.25-0.33) | 0.98 |
| *SCD* | 0.29 (0.26-0.32) |  | 0.28 (0.22-0.32) | 0.43 |
| *SLC25A20* | 0.53 (0.45-0.62) |  | 0.63 (0.52-0.69) | 0.19 |
| *SORL1* | 5.66 (5.23-6.48) |  | 6.47 (5.82-7.23) | 0.13 |
| *SORT1* | 0.59 (0.52-0.74) |  | 0.61 (0.52-0.73) | 0.76 |
| *SREBF1* | 1.01 (0.93-1.09) |  | 1.10 (0.98-1.19) | 0.10 |
| *SREBF2* | 2.90 (2.47-3.21) |  | 3.84 (3.43-4.0) | **0.001** |
| *VLDLR* | 0.02 (0.01-0.03) |  | 0.02 (0.01-0.03) | 0.62 |

Data are presented as median (25^th^ – 75^th^ percentile) of fold change from reference genes (ACSL3 and ACSL4) **(**2^-∆^*^CT^***)** of individual means from the two visits. *P*-values from Mann-Whitney test. h, hour; FH, familial hypercholesterolemia; C_T_, cycle threshold; for gene abbreviations see Table 1.

**Supplementary Table 2.** Postprandial changes in gene expression in peripheral blood mononuclear cells.

| **Target gene** | **n** | **SFA** | |  | **PUFA** | | ***p*_meal_** | ***p*_group_** |
| --- | --- | --- | --- | --- | --- | --- | --- | --- |
|  |  | **4 h (2^-∆∆^*^CT^*)** | **6 h (2^-∆∆^*^CT^*)** |  | **4 h (2^-∆∆^*^CT^*)** | **6 h (2^-∆∆^*^CT^*)** |  |  |
| ***ACACA*** | 82 |  |  |  |  |  | 0.35 | 0.07 |
| FH |  | 1.40 (0.13) | 1.39 (0.07) |  | 1.31 (0.13) | 1.23 (0.13) |  |  |
| C |  | 1.21 (0.16) | 0.98 (0.11) |  | 1.09 (0.07) | 0.96 (0.10) |  |  |
| ***ACAT1*** | 98 |  |  |  |  |  | 0.89 | 0.29 |
| FH |  | 1.27 (0.07) | 1.02 (0.08) |  | 1.35 (0.05) | 1.23 (0.10) |  |  |
| C |  | 1.27 (0.11) | 1.06 (0.11) |  | 1.13 (0.10) | 0.98 (0.11) |  |  |
| ***ACSL1*** | 105 |  |  |  |  |  | 0.67 | 0.73 |
| FH |  | 0.90 (0.05) | 0.98 (0.11) |  | 0.86 (0.05) | 0.89 (0.06) |  |  |
| C |  | 0.94 (0.06) | 0.94 (0.07) |  | 0.93 (0.08) | 0.92 (0.11) |  |  |
| ***CD36*** | 103 |  |  |  |  |  | 0.23 | 0.75 |
| FH |  | 0.92 (0.08) | 0.97 (0.08) |  | 0.97 (0.08) | 1.05 (0.08) |  |  |
| C |  | 0.82 (0.05) | 1.09 (0.10) |  | 1.06 (0.09) | 1.05 (0.06) |  |  |
| ***CPT2*** | 91 |  |  |  |  |  | 0.94 | 0.59 |
| FH |  | 1.02 (0.07) | 1.00 (0.13) |  | 1.09 (0.10) | 1.02 (0.07) |  |  |
| C |  | 1.13 (0.08) | 1.19 (0.09) |  | 1.04 (0.11) | 1.14 (0.11) |  |  |
| ***DHCR24*** | 95 |  |  |  |  |  | 0.23 | 0.44 |
| FH |  | 1.22 (0.10) | 1.05 (0.09) |  | 1.15 (0.07) | 0.98 (0.06) |  |  |
| C |  | 0.98 (0.09) | 0.80 (0.11) |  | 1.15 (0.09) | 1.13 (0.11) |  |  |
| ***DHCR7*** | 98 |  |  |  |  |  | 0.25 | 0.44 |
| FH |  | 1.04 (0.09) | 0.85 (0.04) |  | 1.03 (0.06) | 0.93 (0.07) |  |  |
| C |  | 0.92 (0.05) | 0.81 (0.07) |  | 0.97 (0.05) | 0.93 (0.06) |  |  |
| ***ECI1*** | 104 |  |  |  |  |  | 0.28 | 0.73 |
| FH |  | 1.26 (0.09) | 1.08 (0.10) |  | 1.03 (0.07) | 0.93 (0.06) |  |  |
| C |  | 1.15 (0.09) | 1.04 (0.09) |  | 1.22 (0.11) | 0.99 (0.09) |  |  |
| ***FDFT1*** | 95 |  |  |  |  |  | 0.94 | 0.15 |
| FH |  | 1.80 (0.22) | 1.32 (0.24) |  | 1.55 (0.15) | 1.38 (0.21) |  |  |
| C |  | 1.25 (0.13) | 1.04 (0.10) |  | 1.34 (0.13) | 1.27 (0.17) |  |  |
| ***FDPS*** | 103 |  |  |  |  |  | 0.77 | 0.25 |
| FH |  | 1.32 (0.12) | 1.23 (0.16) |  | 1.38 (0.15) | 1.20 (0.10) |  |  |
| C |  | 1.23 (0.12) | 1.06 (0.10) |  | 1.15 (0.15) | 0.95 (0.11) |  |  |
| ***HMGCR*** | 97 |  |  |  |  |  | 0.23 | 0.43 |
| FH |  | 1.10 (0.07) | 1.18 (0.08) |  | 1.17 (0.08) | 1.15 (0.12) |  |  |
| C |  | 1.00 (0.05) | 1.06 (0.08) |  | 1.15 (0.06) | 1.17 (0.07) |  |  |
| ***LSS*** | 99 |  |  |  |  |  | 0.15 | 0.11 |
| FH |  | 1.06 (0.06) | 1.10 (0.10) |  | 1.16 (0.08) | 1.12 (0.08) |  |  |
| C |  | 1.93 (0.06) | 0.83 (0.03) |  | 1.11 (0.11) | 0.93 (0.06) |  |  |
| ***MYLIP*** | 94 |  |  |  |  |  | 0.15 | 0.59 |
| FH |  | 1.18 (0.09) | 1.05 (0.10) |  | 0.96 (0.11) | 0.87 (0.10) |  |  |
| C |  | 1.04 (0.10) | 0.95 (0.06) |  | 0.93 (0.07) | 0.94 (0.11) |  |  |
| ***NR1H3*** | 92 |  |  |  |  |  | 0.94 | 0.29 |
| FH |  | 1.27 (0.07) | 1.04 (0.09) |  | 1.13 (0.05) | 1.11 (0.10) |  |  |
| C |  | 1.14 (0.09) | 0.93 (0.07) |  | 1.13 (0.07) | 0.99 (0.10) |  |  |
| ***SCAP*** | 98 |  |  |  |  |  | 0.89 | 0.25 |
| FH |  | 1.26 (0.10) | 1.11 (0.10) |  | 1.27 (0.12) | 1.16 (0.11) |  |  |
| C |  | 1.16 (0.11) | 0.99 (0.07) |  | 1.14 (0.10) | 1.00 (0.06) |  |  |
| ***SCARB1*** | 106 |  |  |  |  |  | 0.35 | 0.73 |
| FH |  | 1.01 (0.05) | 1.00 (0.06) |  | 1.11 (0.09) | 1.05 (0.06) |  |  |
| C |  | 1.00 (0.08) | 0.96 (0.08) |  | 1.02 (0.10) | 1.05 (0.11) |  |  |
| ***SCD*** | 91 |  |  |  |  |  | 0.84 | 0.26 |
| FH |  | 1.05 (0.10) | 0.83 (0.06) |  | 1.04 (0.12) | 0.85 (0.08) |  |  |
| C |  | 1.06 (0.08) | 1.15 (0.12) |  | 1.03 (0.09) | 1.06 (0.11) |  |  |
| ***SLC25A20*** | 101 |  |  |  |  |  | 0.74 | 0.55 |
| FH |  | 1.27 (0.15) | 1.46 (0.15) |  | 1.40 (0.14) | 1.26 (0.09) |  |  |
| C |  | 1.16 (0.16) | 1.21 (0.12) |  | 1.24 (0.06) | 1.42 (0.09) |  |  |
| ***SORL1*** | 97 |  |  |  |  |  | 0.51 | 0.29 |
| FH |  | 1.16 (0.09) | 1.18 (0.06) |  | 1.36 (0.07) | 1.30 (0.07) |  |  |
| C |  | 1.26 (0.13) | 1.06 (0.07) |  | 1.16 (0.12) | 1.11 (0.08) |  |  |
| ***SORT1*** | 94 |  |  |  |  |  | 0.15 | 0.75 |
| FH |  | 1.17 (0.12) | 0.98 (0.12) |  | 1.30 (0.15) | 1.17 (0.13) |  |  |
| C |  | 1.07 (0.08) | 1.16 (0.10) |  | 1.27 (0.10) | 1.25 (0.09) |  |  |
| ***SREBF1*** | 88 |  |  |  |  |  | 0.74 | 0.12 |
| FH |  | 1.17 (0.09) | 1.32 (0.10) |  | 1.34 (0.10) | 1.06 (0.06) |  |  |
| C |  | 1.00 (0.09) | 1.12 (0.13) |  | 1.06 (0.09) | 0.98 (0.12) |  |  |
| ***VLDLR*** | 94 |  |  |  |  |  | 0.82 | 0.29 |
| FH |  | 1.42 (0.19) | 1.49 (0.18) |  | 1.41 (0.16) | 1.39 (0.14) |  |  |
| C |  | 1.25 (0.11) | 1.08 (0.15) |  | 1.21 (0.18) | 1.40 (0.14) |  |  |

Data are presented as mean (standard error) of fold change from baseline (0 h) and reference genes (ACSL3 and ACSL4) (2^-∆∆CT^). Benjamini-Hochberg adjusted p-values from a linear mixed model. *n*, number of subjects*2 meals*2 times (4 and 6 h); h, hour; C_T_, cycle threshold; *p*_meal_, *p*-value for change after the SFA vs. PUFA meal; *p*_group_, *p*-value for change in FH vs. control subjects; FH, familial hypercholesterolemia; C, control; *ACACA*, acetyl-CoA carboxylase alpha; *ACAT1,* acetyl-CoA acetyltransferase 1*; ACSL1,* acyl-CoA synthetase long-chain family member 1*; CD36,* CD 36 molecule*; CPT2,* carnitine palmitoyltransferase 2*; DHCR24,* 24-dehydrocholesterol reductase*; DHCR7,* 7-dehydrocholesterol reductase*; ECI1,* enoyl-CoA delta isomerase 1*; FDFT1,* squalene synthase*; FDPS,* farnesyl diphosphate synthase*; HMGCR,* 3-hydroxy-3methylglutaryl-CoA reductase*; LSS,* lanosterol synthase*; MYLIP,* myosin regulatory light chain interacting protein*; NR1H3,* nuclear receptor subfamily 1 group H member 3*; SCAP,* sterol regulatory element binding cleavage activating protein*; SCARB1,* scavenger receptor class B member 1*; SCD,* stearoyl-CoA desaturase*; SLC25A20,* solute carrier family 25 member 20*; SORL1,* sortilin related receptor 1*; SORT1,* sortilin-1*; SREBF1,* sterol regulatory element binding transcription factor 1*; VLDLR,* very low density lipoprotein receptor.
